# Species-Specific Color Preferences During Foraging in *Aedes aegypti*, *Aedes albopictus*, and *Culex quinquefasciatus* Across Varying Light Conditions

**DOI:** 10.3390/insects17030276

**Published:** 2026-03-03

**Authors:** Fanny Hellhammer, Hella Heidtmann, Fritjof Freise, Stefanie C. Becker

**Affiliations:** 1 Research Group for Vector-Associated Biodiversity and Infection, University of Veterinary Medicine Hannover, Buenteweg 17, 30559 Hannover, Germany; 2 Research Center for Emerging Infections and Zoonoses, University of Veterinary Medicine Hannover, Buenteweg 17, 30559 Hannover, Germany; 3 Department of Biometry, Epidemiology and Information Processing, University of Veterinary Medicine Hannover, Buenteweg 2, 30559 Hannover, Germany; fritjof.freise@tiho-hannover.de

**Keywords:** colors, mosquito, foraging, ink, *Aedes*, *Culex*

## Abstract

Mosquitoes are key vectors of numerous infectious diseases, making the study of their behavior essential for effective control strategies. This study investigates the color preferences of *Aedes aegypti*, *Aedes albopictus*, and *Culex quinquefasciatus* during foraging, using an ink-based staining method to assess feeding behavior under varying light intensities (0, 130 and 1600 lx). At 0 lx, no consistent visual preferences emerged, confirming reliance on olfactory cues only. Under dusk-like illumination (130 lx), diurnal *Aedes* exhibited a tendency to approach red stimuli (probably perceived as grey) over darker targets, with *Ae. albopictus* females and males showing significant preference for red over green responses, indicating early salience of red contrasts. At high illumination (1600 lx), *Aedes* shifted preference toward black, especially in males, reflecting dominance of achromatic contrast and camouflage considerations. In contrast, crepuscular *Cx. quinquefasciatus* showed strong attraction to black at dusk-like light in both sexes; at high illumination, females’ preferences shifted from black to red, whereas males maintained or reverted to black preference across assays. These divergent patterns align with differences in photoreceptor sensitivity, contrast processing, and ecological niches governing host- and swarm-seeking. Identifying how dusk-like versus bright light modulates color-driven behavior provides insights for designing trap colors and illumination regimes optimized for specific mosquito species and sexes, thereby enhancing targeted vector-control strategies.

## Simple Summary

Mosquitoes are responsible for spreading many serious diseases, making it important to understand their behavior. In this study, we looked at how three mosquito species (yellow fever mosquito, Asian tiger mosquito, and southern house mosquito) respond to different colors while searching for food. Using a simple method of coloring food with ink, we observed how the mosquitoes reacted to food under different light conditions, like daytime and low light. In darkness, mosquitoes showed no clear color preference, relying only on smell. In dim light, the *Aedes* species started to prefer lighter colors, especially red, likely because it stood out more. This was most noticeable in Asian tiger mosquito males and females. In bright light, these mosquitoes switched to preferring black, possibly because of stronger contrast or camouflage. The *Culex* species behaved differently: both sexes preferred black at dusk-like light, but females switched to red in bright light, while males kept preferring black. These findings show that mosquitoes react differently to color depending on the light level, their species and sex. Knowing this can help improve traps and control strategies tailored to each mosquito type.

## 1. Introduction

As vectors of many infectious diseases, mosquitoes’ interactions with environmental cues, such as color, are of particular interest for optimizing control measures aimed at reducing their population and disease transmission. Visual cues are known to guide mosquitoes in various behaviors, including host-seeking, mate-finding, and oviposition site selection [1,2,3]. This understanding can be used to design more efficient tools for vector control, such as targeted traps or optimized ovitraps. The potential for using visual stimuli, like color, to influence mosquito behavior has been explored in previous studies, demonstrating that mosquitoes exhibit distinct responses to different wavelengths of light [4,5].

In our previous study with *Culex (Cx.) pipiens* biotype *molestus* mosquitoes, we demonstrated that ink-sugar water mixtures provide a cost-effective and simple method for assessing mosquito color preferences [6]. This study aims to expand this approach to additional mosquito species, including the closely related *Cx. quinquefasciatus*, as well as the globally significant disease vectors *Aedes (Ae.) aegypti* (Yellow fever mosquito) and *Ae. albopictus* (Asian tiger mosquito). These species are of particular interest due to their role in transmitting a wide range of viral diseases, including West Nil fever, dengue, Zika, chikungunya, and yellow fever [7,8]. Understanding the visual preferences of these mosquitoes could lead to the development of more effective, targeted control measures that address both population density and the risk of disease transmission, for example, by using colored traps with highly attractive hues to reduce mosquito population density and, consequently, the risk of disease transmission.

The visual systems of mosquitoes are highly adapted to their ecological niches. *Culex pipiens* species, for example, are crepuscular and nocturnal feeders, meaning their visual systems are tuned to low-light environments [9]. Studies suggest that these mosquitoes are less sensitive to color compared to diurnal species, with their visual systems optimized for detecting contrasts rather than distinguishing between a wide range of colors [10]. However, some *Culex* species, including *Cx. pipiens* biotype *molestus*, have been shown to exhibit color preferences when it comes to selecting oviposition sites. For example, black, blue, and red colors have been found to attract these mosquitoes more than other hues [6]. This finding suggests that *Culex* mosquitoes are capable of distinguishing between specific colors, even in dim light. In contrast, mosquitoes of the *Aedes* genus, such as *Ae. aegypti* and *Ae. albopictus*, have different foraging and oviposition behaviors. These species are primarily active in the day and, as a result, their color vision systems are more attuned to the vibrant, saturated hues found in daylight [11].

In our study, we aim to explore and compare the color preferences of these mosquito species by using ink-sugar water mixtures. This method allows us to track the mosquitoes’ color preferences in the context of nectar- and sugar-feeding behavior. We hypothesized that these preferences may reveal species-specific variations in visual attraction. By expanding this approach to include not only *Cx. pipiens* biotype *molestus* [6] but also *Cx. quinquefasciatus*, *Ae. aegypti* and *Ae. albopictus*, we seek to establish a broader understanding of how mosquitoes respond to visual stimuli in different environmental contexts. This expanded knowledge could contribute to the development of more targeted and cost-effective vector control tools that incorporate species-specific visual preferences. By refining and broadening our approach to studying mosquitoes’ color preferences, we aim to provide deeper insight into mosquito–environment interactions, thereby informing the optimization of existing control methods and the design of novel, scalable strategies applicable across diverse ecological and epidemiological settings.

## 2. Materials and Methods

### 2.1. Mosquito Rearing

In this study, three mosquito species were examined: *Cx. quinquefasciatus* (southern house mosquito), *Ae. albopictus* (Asian tiger mosquito), and *Ae. aegypti* (African yellow fever mosquito). The *Cx. quinquefasciatus* mosquitoes (Say, 1823)originated from Malaysia, with the laboratory strain provided by Bayer (Leverkusen, Germany). The *Ae. albopictus* mosquitoes (Skuse, 1894) originated from Nice, France (strain: ALNICE; laboratory colony established in 2011), and the *Ae. aegypti* mosquitoes were sourced from Paea, Tahiti (strain: PAEA; laboratory colony established in 1994). Both *Aedes* strains were supplied by Infravec2.

All three strains were maintained at 28 °C (±1 °C), with a relative humidity of 60–75% and a 16:8 h light:dark photoperiod, including one hour each of simulated dusk and dawn transitions. Larvae from all species were reared in plastic trays or basins filled with dechlorinated tap water. The larvae were fed Tetra Pleco fish food tablets (Tetra Werke, Melle, Germany) until pupation. The emerged adult mosquitoes were housed in cages (BugDorm-1; 30 cm × 30 cm × 30 cm; Bioquip, Compton, CA, USA) and provided ad libitum access to an 8% fructose solution. The fructose solution was supplemented with 0.5 g/L of 4-aminobenzoic acid (PABA) as a dietary additive. Adult mosquitoes were fed animal blood once a week.

### 2.2. Staining Methods/Colors

In accordance with the publication by Hellhammer et al. (2022) [6], which tested the same experiments on *Cx. pipiens* biotype *molestus*, the experiments utilized inks in the colors Strawberry red (hereafter referred to as “red”), Arctic blue (hereafter “blue”), Grass green (hereafter “green”), and Panther black (hereafter “black”) (Seitz-Kreuznach, Bad Kreuznach, Germany). All inks were used at a standardized concentration of 21.5 mL/L of in 8% fructose solution.

### 2.3. Light Intensity

The climate chambers used in the experiments are windowless, eliminating exposure to natural light. They are equipped with fluorescent tubes (Osram T8 Lumilux 58W—865 Daylight White; Osram GmbH, Munich, Germany). The light intensity inside the cages was measured using a luxmeter (Urceri, Shenzhen, China). Experiments were conducted under light conditions of up to 1600 lx, corresponding to the light-dark cycle used during mosquito rearing, as well as at constant 130 lx and darkness (0 lx).

### 2.4. Experimental Design Two-Choice Foraging Assay

The experiments were performed in BugDorm-1 cages (30 cm × 30 cm × 30 cm) under the same climate conditions as those used during mosquito rearing. Approximately 100 non-blood-fed mosquitoes (both sexes), up to seven days old, were used for each experiment. The exact number of *Ae. albopictus*, *Ae. aegypti*, and *Cx. quinquefasciatus* mosquitoes per replicate was determined only after the experiments concluded to avoid disturbances caused by anesthetic measures such as carbon dioxide or coldness, which could affect mosquito behavior and perception. Prior to the experiments, mosquito numbers were estimated, resulting in some variation in group sizes and sex ratios. Consequently, percentage-based analyses were used to ensure comparability between experiments.

The experiments were conducted across multiple mosquito generations and spanned several months. The colors red, blue, green, and black were tested in the same binary combinations as in the study by Hellhammer et al. (2022) [6]: red-blue, red-green, and red-black. However, unlike the referenced study, a comprehensive screening involving all four colors simultaneously was not conducted, as this approach previously resulted in ambiguous mixed-color uptake that limited analytical resolution. Each color combination was tested under three light intensity conditions: constant 0 lx, constant 130 lx, and a normal diurnal rhythm with a maximum intensity of 1600 lx. Each test condition was replicated three times.

**Figure 1:**
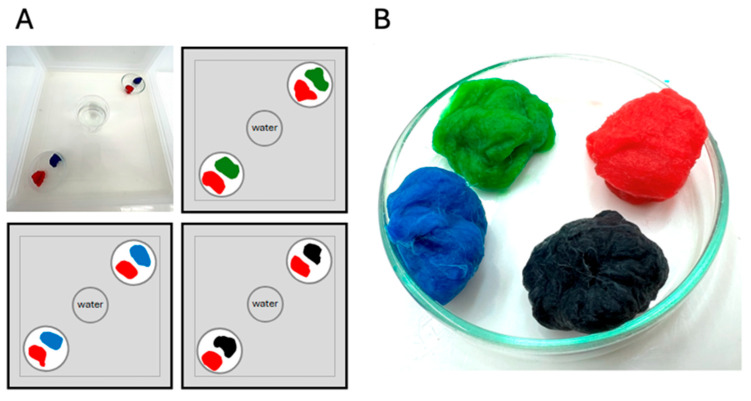
Experimental assay setup used to assess mosquitoes’ color preferences during sugar feeding. (**A**) Photograph (**top left**) and schematic illustration of the assay conducted in a BugDorm-1 cage. The three different color combinations are depicted, as well as the positioning of ink–sugar–soaked cotton pads in glass petri dishes. Each color was offered twice in separate petri dishes. Dechlorinated tap water is centrally provided in a 100-mL glass beaker; (**B**) Close-up photograph of soaked cotton pads, representing all four colors used in this assay—green, blue, black and red.

For the experiments, colored sugar-water solutions were prepared by mixing 21.5 mL/L of ink into 8% fructose solution. These solutions were offered to the mosquitoes in duplicates via soaked cotton pads. Additionally, sugar-free, uncolored, dechlorinated tap water was made available to the mosquitoes. To ensure experimental consistency, identical amounts of cotton were used for all pads, resulting in no differences in surface area or visual appearance. Likewise, equal volumes of liquid were applied to each cotton pad to prevent variation in moisture content or drying rates. The position of the petri dishes and pads within the cages was standardized such that identical colors were oriented toward the same cage corner. Thereby potential biases related to flight distance, cage geometry, or visual field and colored pad position were avoided and to positional effects were minimized, particularly given the predominantly wall-resting behavior of mosquitoes (Figure 1). Additionally, the uncolored water source was always in equal distance to both petri dishes.

Mosquitoes were maintained in cages with continuous access water or to the colored fructose solution for 44–48 h, during which they could voluntarily feed or remain unfed. After this exposure period, mosquitoes were aspirated and visually examined under a stereomicroscope, including determination of their sex. Following ink-sugar ingestion, the mosquitoes’ abdomens displayed coloration corresponding to the consumed dye. Based on abdominal coloration, mosquitoes were categorized into the following groups: single color (red, blue, green, or black), mixed color (mosquitoes that consumed both colors), and uncolored (unfed or water-fed) mosquitoes. Because abdominal color intensity varied with the amount of ingested dye, all classifications were performed by a single observer across all experiments to ensure consistent categorization, particularly in borderline cases. Mosquitoes that died during the experiment were excluded from analysis.

### 2.5. Statistical Analysis

Statistical analysis was performed using R [12]. For the feeding assay, a multinomial logit model with lighting conditions as a factor modeled the number of mosquitoes with a certain color. All possible combinations of color and sex are expressed by the outcome categories (e.g., “female and red”, “male and blue”). The models were fitted using the VGAM package [13]. Likelihood ratio tests, comparing the full model with a constraint one, were used to test the influence of the sex in the feeding assay, preceding pairwise comparisons. For testing the preference of a color compared to another (one-sided tests) and comparing choices of female and male mosquitoes (two-sided tests) in a *post hoc* analysis, Wald tests for linear combinations of the model parameters were used. These comparisons were conducted with the help of the multcomp package [14]. Bonferroni adjustment was used for tests on preference of a color and difference between sexes separately. The proportion of uncolored mosquitoes was additionally analyzed using the glm procedure and a binomial logistic model with light intensity, species and their interaction as effects. A *post hoc* analysis was done using the multcomp package [14] and Bonferroni adjustment. *p*-values < 0.05 were considered to indicate statistical significance, with ≤0.01 and ≤0.001 denoting increased levels of significance.

## 3. Results

**Figure 2:**
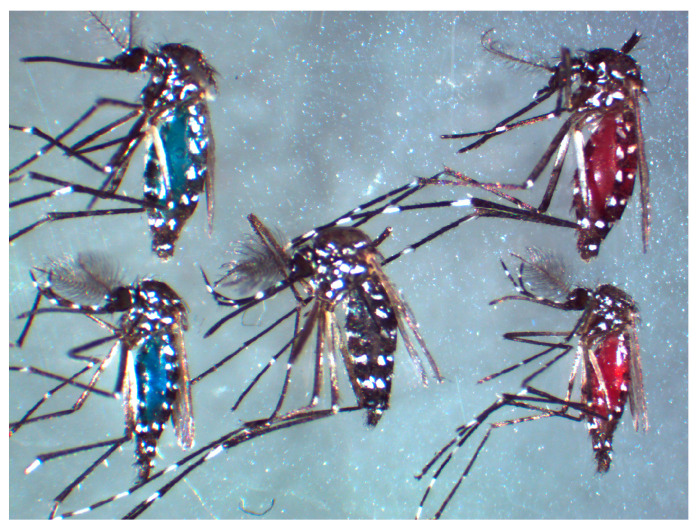
Colored abdomens after feeding assay exemplified using the *Ae. albopictus* mosquito in the “red vs. blue” assay. Top left: blue-colored female; top right: red-colored female; bottom left: blue-colored male; bottom right: red-colored male; center: red-blue mix male.

In the three experimental series “red vs. green,” “red vs. black,” and “red vs. blue,” the color preferences of *Ae. albopictus, Ae. aegypti*, and *Cx. quinquefasciatus* were tested. All species displayed a macroscopically visible stained abdomen after consuming the offered ink solution (Figure 2).

On average, 107 mixed-sex mosquitoes were used per replicate, with the number ranging from 53 to 135 individuals. On average, each trial included 55 females (ranging from 18 to 108) and 52 males (ranging from 17 to 97) (see Table 1 for an overview of the number of mosquitoes used).

**Figure 3:**
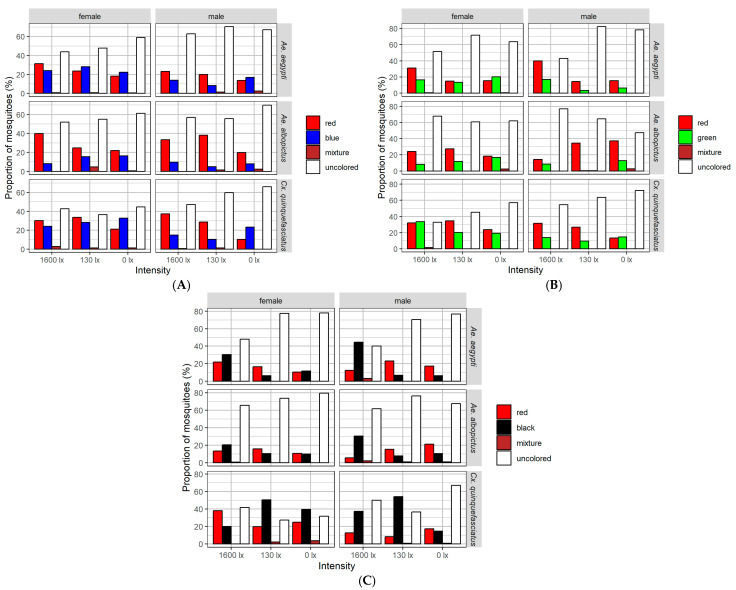
The results of the experiments on color preference in Ae. aegypti, Ae. albopictus, and Cx. quinquefasciatus during feeding were normalized and show the percentage of colored mosquitoes (colored bars) and uncolored mosquitoes (white bars) under light intensities of 0 lx and 130 lx (both constant) and 1600 lx (with a day–-night cycle). The findings are presented as follows: (**A**) results for the red–-blue combination, separated by sex; (**B**) results for the red–-green combination, separated by sex; and (**C**) results for the red–-black combination, separated by sex.

At 1600 lx (with a day-night cycle), an average of 330 (±27) mosquitoes were tested, while at constant 130 lx, the number was 316 (±21), and at constant 0 lx, it was 319 (±25). No significant sex-independent differences in the preference for red, blue, green, and black were observed in these experiments (*p* > 0.05; all results are shown in Figure 3 and Tables S1 and S2).

Significant differences in color preference between red, blue, green and black stimuli were observed in specific species, sexes, and light conditions In the experiments, only mosquitoes with a red coloration were statistically analyzed against their complementary color. Mosquitoes that showed mixed colors, remained unstained, or died during the experiment were excluded from this analysis.

(A) red vs. blue

*Aedes aegypti* males exhibited a significant preference for red over blue at 130 lx (estimate = −0.88, adjusted *p* = 0.03). *Aedes albopictus* females significantly preferred red over blue at 1600 lx (estimate = −1.58, adjusted *p* < 0.001). Males of this species also showed a consistent preference for red across all light conditions: 0 lx (estimate = −0.92, adjusted *p* = 0.04), 130 lx (estimate = −2.06, adjusted *p* < 0.001), and 1600 lx (estimate = −1.24, adjusted *p* = 0.003). *Culex quinquefasciatus* males preferred blue at 0 lx (estimate = 0.81, adjusted *p* = 0.006), whereas at both 130 lx (estimate = −1.02, adjusted *p* < 0.001) and 1600 lx (estimate = −0.92, adjusted *p* < 0.001), a significant preference for red was detected. No other comparison reached statistical significance after correction for multiple testing.

(B) red vs. green

*Aedes aegypti* females showed a significant preference for red over green at 1600 lx (estimate = −0.63, adjusted *p* = 0.03). In males of the same species, a red preference was detected at both 130 lx (estimate = −1.49, adjusted *p* = 0.001) and 1600 lx (estimate = −0.85, adjusted *p* = 0.002). *Aedes albopictus* females preferred red at 130 lx (estimate = −0.84, adjusted *p* = 0.02) and 1600 lx (estimate = −1.07, adjusted *p* = 0.003). Males of this species also exhibited a red preference at 0 lx (estimate = −1.07, adjusted *p* = 0.002) and 130 lx (estimate = −4.19, adjusted *p* < 0.001). For *Cx. quinquefasciatus*, females significantly preferred red at 130 lx (estimate = −0.53, adjusted *p* = 0.04). Males of this species showed a red preference at both 130 lx (estimate = −1.03, adjusted *p* = 0.001) and 1600 lx (estimate = −0.81, adjusted *p* = 0.001). No other conditions showed significant differences after correction for multiple comparisons.

(C) red vs. black

*Aedes aegypti* females significantly preferred red over black at 130 lx (estimate = −0.99, adjusted *p* = 0.02). Males of this species also preferred red at 0 lx (estimate = −1.03, adjusted *p* = 0.02) and 130 lx (estimate = −1.23, adjusted *p* < 0.001). In contrast, black was preferred at 1600 lx in *Ae. aegypti* males (estimate = 1.30, adjusted *p* < 0.001). In *Cx. quinquefasciatus* females, black was significantly preferred at 0 lx (estimate = 0.46, adjusted *p* = 0.04) and 130 lx (estimate = 0.94, adjusted *p* < 0.001), while red was preferred at 1600 lx (estimate = −0.65, adjusted *p* = 0.04). Among *Cx. quinquefasciatus* males, black was preferred at both 130 lx (estimate = 1.86, adjusted *p* < 0.001) and 1600 lx (estimate = 1.08, adjusted *p* < 0.001). No significant preferences were detected for *Ae. albopictus* under any condition following correction for multiple comparisons.

**Figure 4:**
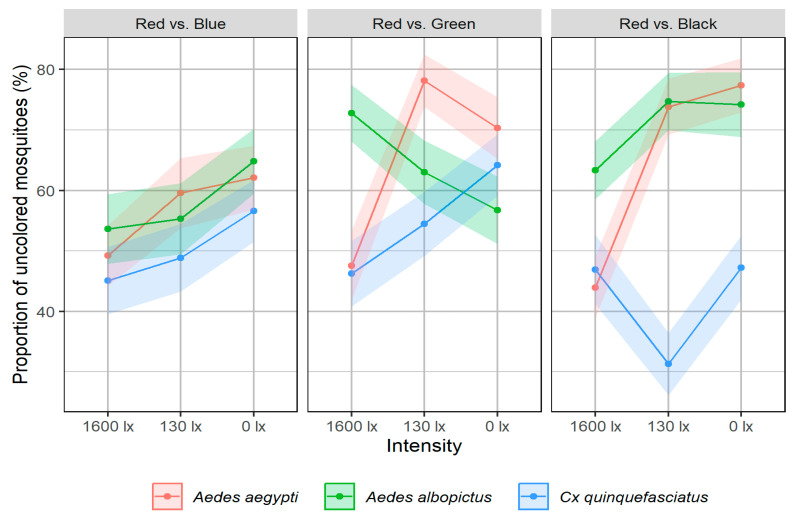
Comparison of the mean percentage of uncolored mosquitoes under different light intensities. Displayed are the mean values for the mosquito species *Ae. aegypti*, *Ae. albopictus*, and *Cx. quinquefasciatus*, along with pointwise 95%-confidence intervals represented as shading.

The investigation of the proportion of uncolored mosquitoes in relation to light intensity and mosquito species revealed the following statistically significant main effects: In all assays, light intensity (*p* < 0.001) and mosquito species (*p* < 0.002) had a significant impact on the proportion of uncolored mosquitoes (Figure 4). In the assays comparing red with green or black a significant interaction was observed (*p* < 0.001). Post hoc tests showed that the significant effect of light intensity was particularly observed at 1600 lx compared to 0 lx (*p* < 0.01). Regarding mosquito species, the significance was driven by the difference between *Cx. quinquefasciatus* and the other two species (*p* < 0.001).

On average, 59.0 ± 4.3% of mosquitoes remained uncolored across all trials. *Cx. quinquefasciatus* showed the lowest values (48.7 ± 5.5%), followed by *Ae. albopictus* (64.1 ± 0.5%) and *Ae. aegypti* (64 ± 8.8%). The highest proportion of uncolored mosquitoes was observed at 0 lx (mean of 63.8%). At 1600 lx, *Cx. quinquefasciatus* and *Ae. aegypti* reached the lowest values (44.8% and 51.5%, respectively). For *Ae. albopictus*, the difference was minimal (Δ < 1.1%).

On average, 62.4% of male mosquitoes remained uncolored, whereas only 55.4% of females did. At all light intensities, fewer uncolored mosquitoes were observed in female *Cx. quinquefasciatus* (on average 40%) compared to males (on average 57.5%). For the two *Aedes* species, the differences between sexes were less pronounced (average Δ < 5%).

## 4. Discussion

The present study investigated the food-color preferences of the mosquito species *Ae. aegypti*, *Ae. albopictus*, and *Cx. quinquefasciatus* under varying light conditions, building on the experimental framework of Hellhammer et al. (2022) [6]. Distinct species- and sex-specific color preferences associated with sugar feeding were observed, with strong modulation by light intensity and color contrast.

In this study, *Cx. quinquefasciatus* displayed pronounced light-intensity-dependent shifts in color preference, consistent with its crepuscular activity profile. Crepuscular and nocturnal species such as *Cx. quinquefasciatus* exhibit a pronounced preference for dark-colored stimuli—a pattern that contrasts with diurnally active mosquitoes and reflects distinct biological and ecological adaptations. Specifically, crepuscular mosquitoes possess larger ommatidia and increased photoreceptor sensitivity, optimizing photon capture under low-light conditions [9]. Dark objects, by minimizing luminance contrast with the ambient environment, afford superior camouflage against nocturnal predators and reduce detection risk. From an evolutionary standpoint, this enhanced attraction to low-reflectance surfaces would confer a selective advantage by improving predator avoidance. In addition, such preferences could facilitate effective navigation toward suitable resting, nectar feeding or host-seeking sites in dimly illuminated habitats.

Under dimmed light illumination (130 lx), both sexes exhibited a robust attraction to black stimuli, suggesting that high-contrast, low-reflectance cues become especially salient in dim environments. At very high illumination (1600 lx), however, sex-specific reversals emerged: females switched their preference from black to red in the red–black assay, whereas males maintained their preference for black. Moreover, at 1600 lx under a simulated photoperiod, males also exhibited a statistically significant preference for red over green, indicating that long-wavelength stimuli can become salient in bright conditions when contrast demands are lower.

These behavioral patterns align with earlier findings that dark hues dominate the visual ecology of *Cx. pipiens* [11]. Allan et al., 1987 [11] first documented a strong response of both sexes to black substrates, and Wen et al., 1997 [13] confirmed that black and brown objects are significantly more attractive than blue, yellow, skin-colored, or white targets under natural and UV illumination. Crucially, the absence of significant preferences in no-light trials underscores the necessity of visual input for these effects. Our previous work using *Cx. pipiens* biotype *molestus* similarly demonstrated strong black preference when ink-based color stimuli were presented [6], reinforcing the conclusion that dark, high-contrast surfaces serve as reliable orientation and landing cues for this genus.

Mechanistically, the enhanced preference for black under crepuscular light conditions may reflect a shift toward luminance-based processing when photon flux is limited. In such contexts, black objects can provide strong achromatic contrast relative to lighter or heterogeneous ambient backgrounds, thereby enhancing detectability under low-light conditions. This effect, however, is inherently background-dependent and may be attenuated in uniformly dark environments. In contrast, males appear to maintain an emphasis on achromatic channels and high-contrast cues (black), possibly because males rely on landmark-based swarm aggregation and conspecific recognition rather than host cues.

For *Ae. albopictus* and *Ae. aegypti*, there are numerous studies examining various aspects of their visual perception, including electroretinograms [15,16], eye morphology [17], the visual components of host-seeking behavior [18], nectar location determination [19], resting behavior [20], oviposition behavior [21,22], and selection of overwintering sites [11]. Our results show that in both *Ae. aegypti* and *Ae. albopictus*, moderate illumination (130 lx) elicited a tendency—occasionally reaching significance—for mosquitoes to approach red stimuli more often than black, whereas under very bright conditions (1600 lx, simulating a day–night cycle), this preference inverted, with black becoming the dominant attractant, particularly in males, where the effect was highly significant. Electrophysiological and behavioral data indicate that *Aedes* mosquitoes possess photoreceptor sensitivity in the UV, blue, and green ranges, whereas sensitivity to long wavelengths (red) is minimal; red stimuli are therefore likely perceived as an achromatic or low-saturation signal rather than a true hue [5]. Several previous studies have documented a general preference of *Ae. aegypti* for dark colors, especially black, both during host-seeking and oviposition [23,24]. These preferences are thought to be linked to the ability of dark objects to provide higher visual contrast in natural habitats, aiding in object detection. Additionally, *Ae. aegypti* has been shown to favor surfaces with low-reflectance factors—particularly black—though red was found to be more attractive than many other low-reflection colors [23]. This aligns with our observation that under moderate illumination, red can be preferred over black, possibly because its reflectance under these conditions offers a balanced mid-gray (red) tone that becomes visually salient. Contrasting findings also exist: some studies have found no significant feeding preferences in *Ae. aegypti* across the visible spectrum, particularly in the 350–700 nm range [5], suggesting that color attraction may be strongly influenced by contextual factors such as light intensity, experimental design, or mosquito physiological state. The observed reversal in preference under high illumination may be due to several interplaying factors. Black surfaces generate the highest achromatic contrast against bright backgrounds due to their minimal reflectivity, making them particularly salient at high light intensities. In addition, darker objects offer better visual camouflage in complex, well-lit environments, possibly reducing predation risk during rest or approach. Given that *Aedes* species are diurnal and thus more exposed to visual predators than nocturnal mosquitoes like *Culex*, the role of camouflage and safe landing zones might be especially important in their visual ecology. In this context, it is important to note that the present experiment does not allow a definitive separation between active sugar–water foraging and color-dependent resting with incidental ingestion. However, the observed behavioral patterns are not consistent with classical resting or refuge selection. At the observation time points at the beginning and at the end of the experiments, mosquitoes were predominantly located on the vertical cage walls, and no pronounced aggregation on any colored substrate was observed (not quantified in this study), in contrast to patterns reported in some resting-focused studies [25,26]. Only few individuals were in contact with the feeding pads at these time points.

The significant red vs. green and red vs. blue preferences in both *Ae. aegypti* and *Ae. albopictus* reveal parallels and subtle divergences that likely reflect species- and sex-specific tuning of contrast detection under varying light intensities, superimposed on a shared predisposition for dark-contrast cues. In the red vs. green assays, both species’ females exhibited significant red-over-green attraction under at least one high-light condition: *Ae. aegypti* females show that preference only at 1600 lx, whereas *Ae. albopictus* females showed red-over-green preference at both 130 lx and 1600 lx. This suggests that *Ae. albopictus* females consistently detect and prefer the gray-level contrast generated by red stimuli over that of green across moderate to bright illumination. This pattern indicates reliable visual discrimination of red-derived mid-gray contrasts relative to chromatically perceived green backgrounds under these light conditions, with red-associated contrasts likely standing out more prominently and thereby guiding host-seeking females toward stimuli that resemble the outlines of skin or clothing. Males of *Ae. aegypti* also preferred red over green at 130 lx and 1600 lx, indicating that red-perceived-as-gray cues remain salient across crepuscular-like and bright conditions. In *Ae. albopictus* males, a preference for red over green was observed even at 0 lx—potentially driven by the odor of the ink—and was also present at 130 lx, indicating that as soon as minimal visual input is available, the red-perceived-as-gray stimulus also visually becomes distinguishable and is preferentially selected over green. These findings align with reports that *Ae. aegypti* shows no inherent preference in the 350–700 nm range during feeding or oviposition [5] and lacks clear green–yellow bias in oviposition contexts [4,27], indicating that red selectivity in host-seeking emerges only in specific light environments where contrast matters most. In the red vs. blue assays, *Ae. albopictus* females significantly preferred red over blue at 130 lx, mirroring Jung et al. (2021) [18]. Here again, the perceived gray level of red likely provides stronger contrast than that of blue under moderate illumination. Male *Ae. albopictus* sometimes favored blue over red at 0 lx (potentially olfactory-driven) and 130 lx, perhaps because under certain setups blue stimuli offered marginally higher contrast or fit photoreceptor sensitivity in that context. For *Ae. aegypti*, red vs. blue results are less consistent, reflecting previous observations of no stable feeding preference across 350–700 nm [5]; any tendency toward red attraction appears context- and light-dependent.

From an ecological perspective, these patterns are consistent with resource-oriented behavior, as accessible plant sugars in natural environments are associated with visually conspicuous structures such as flowers or fruits rather than green foliage, which may represent less accessible or less rewarding sugar sources [28,29]. Dedicated follow-up experiments, explicitly separating color-only resting assays from liquid-feeding contexts, will be required to disentangle resting-site selection from resource-associated color cues.

Comparing species, *Ae. albopictus* shows more consistent red preference across sexes and light levels (especially in host-seeking females), whereas *Ae. aegypti* females display red preference primarily at high light, and males show a robust preference for red over green but variable red vs. blue responses. These interspecific differences may stem from subtle variations in photoreceptor sensitivity, behavioral ecology, or reliance on potential olfactory cues when visual input is minimal. The absence of green or blue preference in oviposition-focused studies [4,27] further underscores that host-seeking contexts—and the resulting color contrasts under particular light intensities—critically shape color-driven behavior in these diurnal mosquitoes.

Another aspect of this study is the proportion of unfed mosquitoes. This proportion indicates how many mosquitoes were not foraging for food and did not ingest colored food during the course of the experiment. In addition to species-specific differences, light intensity was strongly associated with food intake. Although overall activity levels were not directly quantified, the observed pattern suggests that illumination may influence feeding motivation, potentially through effects on movement and energy expenditure. *Culex quinquefasciatus* had the highest proportion of colored mosquitoes in these trials. Their peak food intake occurred in the 130 lx constant trials, closely followed by the day–night rhythm trials up to 1600 lx. This species was also the most active in food seeking during the no-light trials compared to the other two species tested, which corresponds to their natural activity peak (crepuscular and nocturnal). *Aedes aegypti* exhibited very low activity both in the no-light trials and at constant 130 lx. In the day-night rhythm trials with a maximum light intensity of 1600 lx, this species showed the highest proportion of colored mosquitoes, corresponding to the physiological and natural activity peak of this diurnal species. It has been described that the responses of *Ae. aegypti* and *Ae. albopictus* depend on light intensity. It was observed that the nocturnal host-seeking activity in both species positively correlated with increasing light intensity [17]. Furthermore, in this study, it was observed that complete darkness during the day deactivated the host-seeking activity of both species, regardless of their increasing flight activity driven by internal circadian rhythms [17]. Interestingly, *Ae. albopictus* exhibited similar activity to *Ae. aegypti* in other studies [17,24], but in this study, their food intake remained consistently low. Since the experiments were conducted over multiple generations and months, seasonal or generation-specific influences can be excluded. One possible explanation for the observed phenomenon could be that the ink as a staining method has lower acceptance by this mosquito species compared to the other two species. Alternatively, *Ae. albopictus* mosquitoes may have responded quickly to the food offerings and ingested the colors early in the experiment but excreted them before the end of the trial, thus appearing as unfed at the time of the assessment. Further studies are needed to better understand the attractiveness of ink for *Ae. albopictus*, whether it is particularly attractive or repellent and to investigate its digestion and excretion dynamics in greater detail.

Taken together, these species- and context-dependent differences in color-driven behavior indicate that visual preferences emerge from the interaction between sensory physiology, light environment, and sugar-feeding ecology rather than represent fixed traits. Beyond their relevance for understanding nectar- and sugar-seeking behavior, the color preferences identified here may therefore also find practical application. In addition to serving as simple and effective markers for feeding assays in the laboratory, they could be exploited to improve applied control strategies such as attractive toxic sugar baits (ATSBs) [30]. Specifically, adapting bait color to target species and ambient light conditions—using dark, low-reflectance substrates for crepuscular mosquitoes or red stimuli to enhance attraction of diurnal *Aedes* species under moderate illumination—may increase bait detectability and feeding probability without modifying bait composition or toxicant dose, thereby enhancing the efficiency of sugar-based mosquito control.

## 5. Conclusions

Our findings reveal that mosquitoes’ color preferences observed in the context of sugar feeding are finely tuned to species- and sex-specific visual ecology and ambient light conditions: diurnal *Aedes* exhibit red-perceived-as-gray attraction under moderate illumination but shift to black under intense light, whereas crepuscular *Culex* show robust dark-stimulus attraction at dim light with sex-dependent reversals at high intensity. These patterns underscore the role of luminance contrast, photoreceptor sensitivity, and nonvisual–visual integration in shaping host- and swarm-seeking behaviors. Recognizing these dynamics can inform the design of more effective light- and color-tailored vector-control interventions.

## Figures and Tables

**Table 1:** The number of mosquitoes used in the experiments investigating color preferences for the species *Ae. aegypti*, *Ae. albopictus*, and *Cx. quinquefasciatus* is presented. The data include the three color preference experiments: “red vs. green,” “red vs. blue,” and “red vs. black,” as well as the breakdown of mosquito species by sex.

Assay	*Ae.* *aegypti*		*Ae.* *albopictus*		*Cx.* *quinquefasciatus*			Total	
	Female	Male	Female	Male		Female	Male	Female	Male
red vs. green	453	491	488	489		467	526	1408	1506
red vs. blue	603	378	503	363		451	536	1557	1267
red vs. black	513	531	501	465		484	462	1498	1458
Total	1569	1400	1492	1317		1402	1524	4463	4231

## Data Availability

Data is contained within the article or Supplementary Material. The original contributions presented in this study are included in the article/Supplementary Material. Further inquiries can be directed to the corresponding authors.

## Supplementary Materials

The following supporting information can be downloaded at https://www.mdpi.com/article/10.3390/insects17030276/s1. Table S1: Results of one-sided Wald tests testing for color preference in the two-choice feeding bioassays: (A) red vs. blue, (B) red vs. green and (C) red vs. black. All assays were tested at three light intensities. The 1600 lx light intensity was the maximal light intensity of a 16:8 h light cycle (Light:Dark; separated by crepuscular periods; daylight 1600 lx) while the 130 lx and the 0 lx bioassay were at constant light intensity. Results are given for females and males separately. Adjusted *p*-values are calculated using Bonferroni adjustment. Table S2: Results of two-sided Wald tests for comparing choices of female and male mosquitoes in the two-choice feeding bioassays: (A) red vs. blue, (B) red vs. green and (C) red vs. black. All assays were tested at three light intensities. The 1600 lx light intensity was the maximal light intensity of a 16:8 h light cycle (Light:Dark; separated by crepuscular periods; daylight 1600 lx) while the 130 lx and the 0 lx bioassay were at constant light intensity. Results are given for every color of each color combination including the mixture of both colors and uncolored mosquitoes separately. Adjusted *p*-values are calculated using Bonferroni adjustment. Table S3: Overview of the number of mosquitoes that exhibited colored abdomens due to ingestion of dyed food during the experiments. The color (red, blue, green, or any mixture thereof) was not differentiated. For each species—(A) Aedes aegypti, (B) Aedes albopictus, and (C) Culex quinquefasciatus—the absolute number of mosquitoes with visible coloration is provided per assay and sex, along with the corresponding percentage in parentheses.
