# Supplementary material for: Species-Specific Color Preferences During Foraging in Aedes aegypti, Aedes albopictus, and Culex quinquefasciatus Across Varying Light Conditions
Source: Insects. 2026 Mar 3;17(3):276. doi: 10.3390/insects17030276 (PMC13027081; doi:10.3390/insects17030276)
Supplement: Supplementary file 1 [file insects-17-00276-s001.zip › Supplementary Table S3 Number of colored mosquitoes.pdf]

Table S3: Overview of the number of mosquitoes that exhibited colored abdomens due to ingestion of dyed food during the experiments. The color (red, blue, green, or any mixture thereof) was not differentiated. For each species—A) *Aedes aegypti*, B) *Aedes albopictus*, and C) *Culex quinquefasciatus*—the absolute number of mosquitoes with visible coloration is provided per assay and sex, along with the corresponding percentage in parentheses.

A) *Aedes aegypti*

Two-Choice Assay	Light Intensity (in Lux)	Total	Female	Male	Assay
**Red vs. blue**	1600	194/382 (50.8%)	154/274 (56.2%)	40/108 (56.2%)	428/981 (43.6%)
**Red vs. blue**	130	112/277 (40.4%)	69/132 (52.3%)	43/145 (52.3%)	
**Red vs. blue**	0	122/322 (37.9%)	81/197 (41.1%)	41/125 (41.1%)	
**Red vs. green**	1600	150/286 (52.4%)	73/151 (48.3%)	77/135 (48.3%)	318/944 (33.7%)
**Red vs. green**	130	76/348 (21.8%)	38/134 (28.4%)	38/214 (28.4%)	
**Red vs. green**	0	92/310 (29.7%)	61/168 (36.3%)	31/142 (36.3%)	
**Red vs. black**	1600	204/364 (56%)	91/175 (52%)	113/189 (52%)	370/1044 (35.4%)
**Red vs. black**	130	90/344 (26.2%)	37/165 (22.4%)	53/179 (22.4%)	
**Red vs. black**	0	76/336 (22.6%)	38/173 (22%)	38/163 (22%)	
**Overall**		1116/2969 (37.6%)	642/1569 (40.9%)	474/1400 (40.9%)	

B) *Aedes albopictus*

Two-Choice Assay	Light Intensity (in Lux)	Total	Female	Male	Assay
**Red vs. blue**	1600	134/289 (46.4%)	94/196 (48%)	40/93 (48%)	363/866 (41.9%)
**Red vs. blue**	130	122/273 (44.7%)	58/129 (45%)	64/144 (45%)	
**Red vs. blue**	0	107/304 (35.2%)	69/178 (38.8%)	38/126 (38.8%)	
**Red vs. green**	1600	94/345 (27.2%)	51/158 (32.3%)	43/187 (32.3%)	347/977 (35.5%)
**Red vs. green**	130	121/327 (37%)	53/135 (39.3%)	68/192 (39.3%)	
**Red vs. green**	0	132/305 (43.3%)	74/195 (37.9%)	58/110 (37.9%)	
**Red vs. black**	1600	143/390 (36.7%)	54/157 (34.4%)	89/233 (34.4%)	290/966 (30%)
**Red vs. black**	130	81/320 (25.3%)	53/202 (26.2%)	28/118 (26.2%)	
**Red vs. black**	0	66/256 (25.8%)	29/142 (20.4%)	37/114 (20.4%)	
**Overall**		1000/2809 (35.6%)	535/1492 (35.9%)	465/1317 (35.9%)	

C) *Culex quinquefasciatus*

Two-Choice Assay	Light Intensity (in Lux)	Total	Female	Male	Assay
**Red vs. blue**	1600	168/306 (54.9%)	83/145 (57.2%)	85/161 (57.2%)	483/977 (49.4%)
**Red vs. blue**	130	158/309 (51.1%)	92/145 (63.4%)	66/164 (63.4%)	
**Red vs. blue**	0	157/362 (43.4%)	89/161 (55.3%)	68/201 (55.3%)	
**Red vs. green**	1600	173/322 (53.7%)	82/122 (67.2%)	91/200 (67.2%)	446/993 (44.9%)
**Red vs. green**	130	153/336 (45.5%)	92/168 (54.8%)	61/168 (54.8%)	
**Red vs. green**	0	120/335 (35.8%)	76/177 (42.9%)	44/158 (42.9%)	
**Red vs. black**	1600	155/292 (53.1%)	64/110 (58.2%)	91/182 (58.2%)	550/946 (58.1%)
**Red vs. black**	130	215/313 (68.7%)	132/182 (72.5%)	83/131 (72.5%)	
**Red vs. black**	0	180/341 (52.8%)	131/192 (68.2%)	49/149 (68.2%)	
**Overall**		1479/2916 (50.7%)	841/1402 (60%)	638/1514 (60%)	
