# Supplementary material for: Species-Specific Color Preferences During Foraging in Aedes aegypti, Aedes albopictus, and Culex quinquefasciatus Across Varying Light Conditions
Source: Insects. 2026 Mar 3;17(3):276. doi: 10.3390/insects17030276 (PMC13027081; doi:10.3390/insects17030276)
Supplement: Supplementary file 1 [file insects-17-00276-s001.zip › Supplementary Table S1 One-sided statistical analysis of the results of the two-choice feeding bioassay_2025-06-11_FF.pdf]

Table S1: Results of one-sided Wald tests testing for color preference in the two-choice feeding bioassays: A) red vs. blue, B) red vs. green and C) red vs. black. All assays were tested at three light intensities. The 1600 lx light intensity was the maximal light intensity of an 16:8 hours light cycle (Light:Dark; separated by crepuscular periods; daylight 1600 lx) while the 130 lx and the 0 lx bioassay were at constant light intensity. Results are given for females and males separately. Adjusted p-values are calculated using Bonferroni adjustment.

A) Red vs. Blue

Species	Lux	Sex	Null hypothesis	Estimate	Std. error	t-statistic	p-value	Adjusted p-value	Significance
Aedes aegypti	0	female	Red ≥ Blue	0,20067072	0,22472389	0,89296568	0,18593778	1	n.s.
Aedes aegypti	130	female	Red ≥ Blue	0,17693071	0,24348743	0,72665233	0,23371948	1	n.s.
Aedes aegypti	1600	female	Red ≤≤ Blue	-0,26469255	0,16364419	-1,61748822	0,05288647	0,31731881	n.s.
Aedes aegypti	0	male	Red ≥ Blue	0,21130909	0,32626077	0,64766932	0,25859943	1	n.s.
Aedes aegypti	130	male	Red ≤≤ Blue	-0,88238918	0,34324778	-2,57070617	0,00507457	0,03044742	*
Aedes aegypti	1600	male	Red ≤≤ Blue	-0,51082562	0,32659862	-1,5640777	0,05889966	0,35339797	n.s.
Aedes albopictus	0	female	Red ≤≤ Blue	-0,296265816	0,24520152	-1,208254402	0,113474711	0,680848266	n.s.
Aedes albopictus	130	female	Red ≤≤ Blue	-0,470003629	0,285043856	-1,648881809	0,049585925	0,297515552	n.s.
Aedes albopictus	1600	female	Red ≤≤ Blue	-1,584120104	0,274445841	-5,772068172	3,91522E-09	2,34913E-08	***
Aedes albopictus	0	male	Red ≤≤ Blue	-0,916290732	0,37416566	-2,448890506	0,00716485	0,042989101	*
Aedes albopictus	130	male	Red ≤≤ Blue	-2,061423036	0,4012966	-5,136906312	1,39649E-07	8,37894E-07	***
Aedes albopictus	1600	male	Red ≤≤ Blue	-1,236762627	0,378640809	-3,266321534	0,000544772	0,003268634	**
Cx quinquefasciatus	0	female	Red ≥ Blue	0,443931389	0,219726402	2,020382552	0,021671861	0,130031166	n.s.
Cx quinquefasciatus	130	female	Red ≤≤ Blue	-0,178248231	0,211656342	-0,842158707	0,199849562	1	n.s.
Cx quinquefasciatus	1600	female	Red ≤≤ Blue	-0,228841572	0,226492166	-1,010373014	0,156158306	0,936949836	n.s.
Cx quinquefasciatus	0	male	Red ≥ Blue	0,805625164	0,262479721	3,069285351	0,001072858	0,006437146	**
Cx quinquefasciatus	130	male	Red ≤≤ Blue	-1,016934258	0,283019655	-3,593157717	0,000163347	0,000980084	***
Cx quinquefasciatus	1600	male	Red ≤≤ Blue	-0,916290732	0,241522946	-3,793804058	7,41784E-05	0,00044507	***

B) Red vs. Green

**Species**	**Lux**	**Sex**	**Null hypothesis**	**Estimate**	**Std. error**	**t-statistic**	**p-value**	**Adjusted p-value**	**Significance**
Aedes aegypti	0	female	Red ≥ Green	0,26826399	0,26052505	1,02970514	0,15157422	0,90944532	n.s.
Aedes aegypti	130	female	Red ≤≤ Green	-0,10536052	0,32489313	-0,32429284	0,37285817	1	n.s.
Aedes aegypti	1600	female	Red ≤≤ Green	-0,63127178	0,2475411	-2,55016956	0,00538353	0,03230116	*
Aedes aegypti	0	male	Red ≤≤ Green	-0,89381788	0,39568378	-2,25891965	0,01194419	0,07166515	n.s.
Aedes aegypti	130	male	Red ≤≤ Green	-1,48807706	0,41846769	-3,5560142	0,00018826	0,00112957	**
Aedes aegypti	1600	male	Red ≤≤ Green	-0,85348983	0,24899152	-3,42778667	0,00030426	0,00182557	**
Aedes albopictus	0	female	Red ≤≤ Green	-0,08701138	0,2409996	-0,36104365	0,35903341	1	n.s.
Aedes albopictus	130	female	Red ≤≤ Green	-0,83832919	0,29921068	-2,80180241	0,0025409	0,0152454	*
Aedes albopictus	1600	female	Red ≤≤ Green	-1,0726368	0,32130806	-3,33834395	0,0004214	0,00252838	**
Aedes albopictus	0	male	Red ≤≤ Green	-1,07451474	0,30954614	-3,47125867	0,00025901	0,00155407	**
Aedes albopictus	130	male	Red ≤≤ Green	-4,18965474	1,00754493	-4,15828082	1,6033E-05	9,6196E-05	***
Aedes albopictus	1600	male	Red ≤≤ Green	-0,52324814	0,31549491	-1,6584995	0,04860834	0,29165007	n.s.
Cx quinquefasciatus	0	female	Red ≤≤ Green	-0,21130909	0,2306974	-0,91595786	0,1798445	1	n.s.
Cx quinquefasciatus	130	female	Red ≤ Green	-0,53408249	0,21599339	-2,47267978	0,00670521	0,04023127	*
Cx quinquefasciatus	1600	female	Red ≥ Green	0,05001042	0,22367671	0,2235835	0,4115407	1	n.s.
Cx quinquefasciatus	0	male	Red ≥ Green	0,09097178	0,3018233	0,3014074	0,38155192	1	n.s.
Cx quinquefasciatus	130	male	Red ≤ Green	-1,03407377	0,29107082	-3,5526535	0,00019068	0,0011441	**
Cx quinquefasciatus	1600	male	Red ≤ Green	-0,81093022	0,22712838	-3,57036057	0,00017825	0,00106947	**

C) Red vs. Black

Species	Lux	Sex	Null hypothesis	Estimate	Std. error	t-statistic	p-value	Adjusted p-value	Significance
Aedes aegypti	0	female	Red ≥ Black	0,105360516	0,324891926	0,324294042	0,372857715	1	n.s.
Aedes aegypti	130	female	Red ≤ Black	-0,993251771	0,370176761	-2,683182404	0,00364626	0,021877561	*
Aedes aegypti	1600	female	Red ≥ Black	0,332705754	0,212564314	1,565200423	0,058767961	0,352607767	n.s.
Aedes aegypti	0	male	Red ≤ Black	-1,029619419	0,368392395	-2,794898679	0,002595799	0,015574795	*
Aedes aegypti	130	male	Red ≤ Black	-1,228665417	0,328213009	-3,743500054	9,07373E-05	0,000544424	***
Aedes aegypti	1600	male	Red ≥ Black	1,295322583	0,23533621	5,504136339	1,85491E-08	1,11295E-07	***
Aedes albopictus	0	female	Red ≤ Black	-0,068992871	0,371611645	-0,185658529	0,426356276	1	n.s.
Aedes albopictus	130	female	Red ≤ Black	-0,421213465	0,280836637	-1,499852263	0,066826338	0,400958028	n.s.
Aedes albopictus	1600	female	Red ≥ Black	0,421213465	0,280836336	1,499853869	0,06682613	0,400956779	n.s.
Aedes albopictus	0	male	Red ≤ Black	-0,693147181	0,353553394	-1,960516267	0,024967739	0,149806436	n.s.
Aedes albopictus	130	male	Red ≤ Black	-0,693147178	0,408238382	-1,697898111	0,044763497	0,268580981	n.s.
Aedes albopictus	1600	male	Red ≥ Black	1,69773052	0,301674633	5,627687369	9,13209E-09	5,47925E-08	n.s.
Cx quinquefasciatus	0	female	Red ≥ Black	0,459532329	0,184367103	2,492485497	0,006342626	0,038055753	*
Cx quinquefasciatus	130	female	Red ≥ Black	0,938269639	0,196589275	4,772740727	9,08678E-07	5,45207E-06	***
Cx quinquefasciatus	1600	female	Red ≤ Black	-0,646627165	0,263180678	-2,456970511	0,007005709	0,042034256	*
Cx quinquefasciatus	0	male	Red ≤ Black	-0,167054085	0,28968272	-0,576679494	0,282077994	1	n.s.
Cx quinquefasciatus	130	male	Red ≥ Black	1,864784604	0,324027156	5,755025678	4,33143E-09	2,59886E-08	***
Cx quinquefasciatus	1600	male	Red ≥ Black	1,084013489	0,241213895	4,493992722	3,495E-06	2,097E-05	***
