# Supplementary material for: Species-Specific Color Preferences During Foraging in Aedes aegypti, Aedes albopictus, and Culex quinquefasciatus Across Varying Light Conditions
Source: Insects. 2026 Mar 3;17(3):276. doi: 10.3390/insects17030276 (PMC13027081; doi:10.3390/insects17030276)
Supplement: Supplementary file 1 [file insects-17-00276-s001.zip › Supplementary Table S2 Two-sided statistical analysis of the results of the two-choice feeding bioassay.pdf]

Table S2: Results of two-sided Wald tests for comparing choices of female and male mosquitoes in the two-choice feeding bioassays: A) red vs. blue, B) red vs. green and C) red vs. black. All assays were tested at three light intensities. The 1600 lx light intensity was the maximal light intensity of an 16:8 hours light cycle (Light:Dark; separated by crepuscular periods; daylight 1600 lx) while the 130 lx and the 0 lx bioassay were at constant light intensity. Results are given for each color of each color combination including the mixture of both colors and uncolored mosquitoes separately. Adjusted p-values are calculated using Bonferroni adjustment.

A) Red vs. Blue

Species	Lux	Color	Null hypothesis	Estimate	Std. error	t-statisitic	p-value	Adjusted p-value	Significance
Aedes aegypti	0	red	female = male	-0,75030559	0,29428619	-2,54957796	0,01078534	0,09706805	n.s.
Aedes aegypti	0	blue	female = male	-0,73966722	0,26522171	-2,78886381	0,00528933	0,04760397	*
Aedes aegypti	0	clear	female = male	0,32277339	0,14326963	2,25290867	0,02426491	0,21838418	n.s.
Aedes aegypti	130	red	female = male	-0,06669137	0,25834558	-0,25814792	0,79629275	1	n.s.
Aedes aegypti	130	blue	female = male	-1,12601126	0,33220886	-3,38946787	0,00070028	0,00630256	**
Aedes aegypti	130	clear	female = male	0,48183808	0,16023723	3,00702952	0,00263814	0,02374327	*
Aedes aegypti	1600	red	female = male	-1,23547147	0,22721775	-5,43738986	5,4067E-08	4,866E-07	***
Aedes aegypti	1600	blue	female = male	-1,48160454	0,28603877	-5,17973327	2,222E-07	1,9998E-06	***
Aedes aegypti	1600	clear	female = male	-0,56798404	0,15178675	-3,74198701	0,00018257	0,00164314	**
Aedes albopictus	0	red	female = male	-0,44468582	0,25620505	-1,73566377	0,0826233	0,74360973	n.s.
Aedes albopictus	0	blue	female = male	-1,06471074	0,36671883	-2,90334352	0,00369201	0,03322813	*
Aedes albopictus	0	clear	female = male	0,21401107	0,14331067	1,49333657	0,13534911	1	n.s.
Aedes albopictus	130	red	female = male	0,54159728	0,22233267	2,435977	0,01485163	0,13366466	n.s.
Aedes albopictus	130	blue	female = male	-1,04982212	0,43915503	-2,39055014	0,01682315	0,15140837	n.s.
Aedes albopictus	130	clear	female = male	0,11934676	0,16304756	0,73197512	0,46418375	1	n.s.
Aedes albopictus	1600	red	female = male	-0,92272162	0,21231717	-4,34595852	1,3867E-05	0,0001248	***
Aedes albopictus	1600	blue	female = male	-0,57536414	0,41666629	-1,38087518	0,16731734	1	n.s.
Aedes albopictus	1600	clear	female = male	-0,6546809	0,16932764	-3,86635584	0,00011047	0,00099426	***
Cx quinquefasciatus	0	red	female = male	-0,48183809	0,27754418	-1,73607705	0,08255021	0,7429519	n.s.
Cx quinquefasciatus	0	blue	female = male	-0,12014431	0,20036098	-0,59963927	0,54874667	1	n.s.

Cx quinquefasciatus	0	clear	female = male	-0,61368301	0,14631366	-4,19429745	2,7372E-05	0,00024635	***
Cx quinquefasciatus	130	red	female = male	-0,0416727	0,20416846	-0,20410938	0,83826803	1	n.s.
Cx quinquefasciatus	130	blue	female = male	-0,88035872	0,28846798	-3,05184213	0,00227442	0,02046975	*
Cx quinquefasciatus	130	clear	female = male	0,61467557	0,17050515	3,60502639	0,00031212	0,00280909	**
Cx quinquefasciatus	1600	red	female = male	0,31015493	0,19847907	1,56265815	0,11813302	1	n.s.
Cx quinquefasciatus	1600	blue	female = male	-0,37729423	0,26502471	-1,42361909	0,15455677	1	n.s.
Cx quinquefasciatus	1600	clear	female = male	0,20359896	0,17113424	1,18970324	0,23416305	1	n.s.

B) Red vs. Green

Species	Lux	Color	Null hypothesis	Estimate	Std. error	t-statisitic	p-value	Adjusted p-value	Significance
Aedes aegypti	0	red	female = male	-0,16705408	0,28968273	-0,57667948	0,564156	1	n.s.
Aedes aegypti	0	blue	female = male	-1,32913595	0,37486381	-3,54565022	0,00039165	0,00352481	**
Aedes aegypti	0	clear	female = male	-0,03670137	0,1354799	-0,27089898	0,78646873	1	n.s.
Aedes aegypti	130	red	female = male	0,43825493	0,28680667	1,52805	0,12650012	1	n.s.
Aedes aegypti	130	blue	female = male	-0,94446161	0,44543539	-2,12031109	0,03397982	0,30581837	n.s.
Aedes aegypti	130	clear	female = male	0,6061358	0,12687981	4,77724412	1,7771E-06	1,5994E-05	***
Aedes aegypti	1600	red	female = male	0,13883644	0,19948713	0,69596693	0,48644954	1	n.s.
Aedes aegypti	1600	blue	female = male	-0,08338161	0,28892605	-0,28859153	0,77289398	1	n.s.
Aedes aegypti	1600	clear	female = male	-0,29626582	0,17338366	-1,70872978	0,08750102	0,78750914	n.s.
Aedes albopictus	0	red	female = male	0,13005313	0,2284032	0,56940152	0,56908369	1	n.s.
Aedes albopictus	0	blue	female = male	-0,85745023	0,31895392	-2,68832011	0,00718125	0,06463127	n.s.
Aedes albopictus	0	clear	female = male	0,84454683	0,16581686	5,09325054	3,5198E-07	3,1678E-06	***
Aedes albopictus	130	red	female = male	0,57873683	0,20537415	2,81796331	0,00483293	0,0434964	*
Aedes albopictus	130	blue	female = male	-2,77258872	1,03077411	-2,68981215	0,00714923	0,06434303	n.s.
Aedes albopictus	130	clear	female = male	0,41356232	0,14233636	2,90552835	0,00366634	0,03299703	*
Aedes albopictus	1600	red	female = male	-0,34174929	0,25169987	-1,35776506	0,17453825	1	n.s.
Aedes albopictus	1600	blue	female = male	0,20763936	0,373394	0,55608651	0,57815171	1	n.s.

Aedes albopictus	1600	clear	female = male	0,29698447	0,12763322	2,32685867	0,01997279	0,1797551	n.s.
Cx quinquefasciatus	0	red	female = male	-0,69314718	0,26726124	-2,59351926	0,00949992	0,0854993	n.s.
Cx quinquefasciatus	0	blue	female = male	-0,39086631	0,26998152	-1,44775207	0,1476864	1	n.s.
Cx quinquefasciatus	0	clear	female = male	-0,12107793	0,13664889	-0,88605132	0,37558988	1	n.s.
Cx quinquefasciatus	130	red	female = male	-0,25378052	0,19865448	-1,27749711	0,2014268	1	n.s.
Cx quinquefasciatus	130	blue	female = male	-0,7537718	0,30316953	-2,48630461	0,01290774	0,11616969	n.s.
Cx quinquefasciatus	130	clear	female = male	0,34209549	0,1500123	2,28044968	0,02258103	0,20322929	n.s.
Cx quinquefasciatus	1600	red	female = male	0,47957308	0,20374995	2,3537335	0,01858593	0,16727337	n.s.
Cx quinquefasciatus	1600	blue	female = male	-0,38136756	0,24516225	-1,55557209	0,11980988	1	n.s.
Cx quinquefasciatus	1600	clear	female = male	1,00246843	0,18486295	5,42276537	5,8684E-08	5,2816E-07	***

C) Red vs. Black

Species	Lux	Color	Null hypothesis	Estimate	Std. error	t-statisitic	p-value	Adjusted p-value	Significance
Aedes aegypti	0	red	female = male	0,44183275	0,3021061	1,46250855	0,1436019	1	n.s.
Aedes aegypti	0	blue	female = male	-0,69314718	0,38729785	-1,78970056	0,07350206	0,66151856	n.s.
Aedes aegypti	0	clear	female = male	0,07696104	0,12412703	0,62001839	0,53524568	1	n.s.
Aedes aegypti	130	red	female = male	0,4177352	0,24784551	1,68546609	0,09189868	0,82708814	n.s.
Aedes aegypti	130	blue	female = male	0,18232155	0,42816728	0,42581852	0,67024009	1	n.s.
Aedes aegypti	130	clear	female = male	-0,01574836	0,12549517	-0,12548975	0,90013584	1	n.s.
Aedes aegypti	1600	red	female = male	-0,50209194	0,26418596	-1,90052471	0,0573643	0,51627866	n.s.
Aedes aegypti	1600	blue	female = male	0,46052489	0,17542092	2,62525641	0,00865837	0,07792536	n.s.
Aedes aegypti	1600	clear	female = male	-0,10008346	0,15831208	-0,63219092	0,52726213	1	n.s.
Aedes albopictus	0	red	female = male	0,47000363	0,3291403	1,42797352	0,15329948	1	n.s.
Aedes albopictus	0	blue	female = male	-0,15415068	0,39339787	-0,39184422	0,69517332	1	n.s.
Aedes albopictus	0	clear	female = male	0,3835824	0,14777202	2,59577143	0,00943788	0,08494094	n.s.
Aedes albopictus	130	red	female = male	-0,57536414	0,29462814	-1,95284857	0,05083754	0,45753787	n.s.
Aedes albopictus	130	blue	female = male	-0,84729786	0,39839936	-2,12675506	0,03344044	0,30096396	n.s.

Aedes albopictus	130	clear	female = male	-0,50413664	0,13350116	-3,77627168	0,00015919	0,00143274	**
Aedes albopictus	1600	red	female = male	-0,47957308	0,35290526	-1,35892867	0,17416919	1	n.s.
Aedes albopictus	1600	blue	female = male	0,79694397	0,21291902	3,74294411	0,00018188	0,00163689	**
Aedes albopictus	1600	clear	female = male	0,33508431	0,12904721	2,59660253	0,00941508	0,08473572	n.s.
Cx quinquefasciatus	0	red	female = male	-0,61310447	0,24350537	-2,5178273	0,01180812	0,10627308	n.s.
Cx quinquefasciatus	0	blue	female = male	-1,23969089	0,24210006	-5,12057232	3,0461E-07	2,7415E-06	***
Cx quinquefasciatus	0	clear	female = male	-0,49429632	0,16246059	-3,04256143	0,00234574	0,02111165	*
Cx quinquefasciatus	130	red	female = male	-1,18562367	0,34450961	-3,44148217	0,00057854	0,00520683	**
Cx quinquefasciatus	130	blue	female = male	-0,2591087	0,15796858	-1,64025466	0,10095223	0,90857005	n.s.
Cx quinquefasciatus	130	clear	female = male	-0,04082199	0,20207259	-0,20201648	0,83990384	1	n.s.
Cx quinquefasciatus	1600	red	female = male	-0,6021754	0,25939889	-2,32142628	0,02026385	0,18237464	n.s.
Cx quinquefasciatus	1600	blue	female = male	1,12846525	0,24527623	4,60079341	4,2088E-06	3,788E-05	***
Cx quinquefasciatus	1600	clear	female = male	0,68221811	0,18090921	3,77105245	0,00016256	0,00146304	**
